# A deterministic approach for design of supervisory control of LPV systems with delay

**DOI:** 10.1371/journal.pone.0256408

**Published:** 2021-08-20

**Authors:** Ch. Nauman Zahid, Mina Salim, Raja Ali Riaz, Jamshed Iqbal

**Affiliations:** 1 Department of Electrical and Computer Engineering, COMSATS University, Islamabad, Pakistan; 2 Department of Control Engineering, Faculty of Electrical & Computer Engineering, University of Tabriz, Tabriz, Iran; 3 Department of Computer Science and Technology, Faculty of Science and Engineering, University of Hull, Hull, United Kingdom; National Huaqiao University, CHINA

## Abstract

Linear Parameter Varying (LPV) systems and their control have gained attraction recently as they approximate nonlinear systems with higher order than ordinary linear systems. On the other hand, time delay is an inherent part of various real-life applications. A supervisory control structure is proposed in this paper for LPV systems subject to time delays. In the proposed control structure, a supervisor selects the most suitable controller from a bank of controllers; which desires to enhance the performance of closed-loop system in contrast with using a single robust controller. The analysis is based on the celebrated Smith predictor for time delay compensation and we provide a sufficient condition to assure the stability of the closed-loop switched system in terms of dwell time. Simulations on blood pressure control of hypertension patients in postoperative scenario are used to exemplify the effectiveness of the utilized technique. The operating region of the system is partitioned into five smaller operating regions to construct corresponding robust controllers and perform hysteresis switching amongst them. Simulation results witnessed that the proposed control scheme demonstrated a pressure undershoot less than the desired value of 10 *mmHg* while the Mean Arterial Pressure (MAP) remains within ±5 *mmHg* of the desired value.

## I Introduction

Time delay appears in various real-world applications such as economics, biology, ecology, chemical processes, and social sciences. It arises naturally in those physical phenomena which involve material transportation or information transmission. This ubiquity has made the study of time delay systems vastly attractive over the past few decades [1–3]. The presence of time delay in system dynamics is often responsible for instability and performance degradation. Motivated by the consequences of time delay, a lot of attention has been given to various issues pertaining to time delay systems, [4, 5]. At the same time, the significance of linear parameter varying (LPV) systems has been pointed out in numerous contributions, [6–8]. Numerous real-life phenomena can be modeled using LPV systems that involve nonlinearities and uncertainties, [9–11]. However, it is worth mentioning that various issues pertaining to LPV framework still remain open with regard to stability analysis and synthesizing control law [12].

The stability analysis of LPV time-delayed systems is intriguing and recently has attracted significant attention of the control community because, (i) these systems are an overlap intersection of two different class of systems and assume difficulties from each of these classes, [13], (ii) many analysis tools such as projection lemma [14], and dualization lemma, [15], which are devised for LPV system analysis fall apart when applied to LPV systems with time delay, and (iii) many results such as frequency domain methods or techniques based upon eigenvalues, which are targeted for linear time invariant systems (LTI) incorporating time delay are not applicable for LPV time-delayed systems owing to their time varying identity. A lot of effort has been made to analyze LPV systems with delay in more novel ways [16–18]. Distinctively [16], employed a Lyapunov—Krasovskii functional dependent on system parameters in combination with Jensen’s inequality. Their aim was to derive stability results, however, this approach results in non-linear matrix inequalities and authors have to employ non-trivial results from literature to form tractable LMIs. Also, literature on control systems reports several gain scheduling algorithms. The control strategy mentioned in [19] involves selecting a reference trajectory with reference to which the operating points are selected. Corresponding to each of these points, a separate controller is designed after linearizing the system. Research work in [17] reported a novel condition for sufficient stability of delay-dependent systems; and the criteria to design gain-scheduling state-feedback control alongwith gain-scheduling static output-feedback. The approach separated the Lyapunov matrices and the system matrices, so that stabilizating controllers can be designed in a new way. Similar Lyapunov- Krasovskii functionals based analysis has been presented in [20–22], nevertheless, they yield results that are computationally expensive and cumbersome to implement. An interesting approach has been studied in [23] where a model transformation is employed to synthesize a delay-scheduled state-feedback for LPV time delay systems in “linear fractional transformation (LFT)” form. Another controller synthesis scheduled by time delay is presented in [24]. The authors have employed an advanced model transformation on to the time-delayed system, thus turning it into a LPV system with uncertainty. Later on, this transformation is used to derive a delay-dependent stability result that relies on full block-procedure. The work in [13], presented the synthesizing of controllers which were resilient when it came to the uncertainties upon the implemented delay. These controllers combine both memoryless and exact-memory controllers. The work in [25] investigated filtering problem for a certain class of LPV systems, whereas authors in [26] derived a reduced order model for switched LPV systems. See also [27] which is dedicated to the study of LPV systems with time delay. Although there is an abundance of promising results, the challenges relating control and stability analysis of LPV systems with time delay still remain sporadic, requiring further explorations [13].

In this article, we present a deterministic approach to supervisory control uncertain LPV time delay systems, whose parameters are scheduled across a measurable trajectory (as an example blood pressure regulation problem will be discussed later in this paper). The works in [28–30] can be referred as the motivation for applying switching in control systems. This control design involves various steps. First, we use the celebrated Smith predictor approach to get rid of time delay from controller synthesis [31]. Secondly, a family of controllers is constructed for the LPV time delay system, with each controller ensuring robust stability for a specific operating range. Then, we carry out hysteresis switching among the robust controllers based on the switching law that relies on the scheduling variable. This will allow our system to function over a larger range of LPV system with time delay. A switching robust control scheme is adopted because in certain scenarios, a single controller would not be sufficient to robustly stabilize LPV system, over the entire range of operation [29, 32, 33]. We provide a sufficient condition in terms of bound on the rate of parameter variation to ensure the stability of the closed-loop switched system. LPV systems with switching control for LPV systems has also been discussed in [32], and [34]. However, no delay is present in [32] while state feedback controllers are proposed in [34] instead of *H*_∞_ controllers. As robust controllers minimize the impact of perturbrations and uncertainty, they are a desired choice over conventional state-feedback control system.

*Novelty*: In almost all of aforementioned works; Lyapunov-Kravoskii analysis has been employed which yields conservatism due to Jensen’s inequality, computationaly expensive, and results in parameter dependent conrollers which are cumbersome to discretize and implement in practical applications. Our approach has the following features:

- It doesn’t employ any Lyapunov-Karavoskii functional; hence we get rid of conservatism
- It utilizes LTI smith-predictor based controllers instead of LPV controllers to reduce complexity
- The controller implementation becomes easier as compared to discretization of LPV controllers [35]
- As there are fewer decision variables for LTI controllers, the computational complexity has reduced considerably
- In our stability analysis, sufficient condition is provided with respect to bound on the rate of parameter variation

In this work, we have provided stability analysis for LPV time delay systems. Here, the delay is incorporated at the input, whereas previous works have considered a time delay in the states.

The remaining document is structured in the following order. Section II provides the description of control problem and its preliminaries. Section III focuses on design of switching robust controllers. Section IV presents the stability result for the overall closed-loop switched system while section V illustrates the application of our proposed method on a practical problem of blood pressure regulation. Section VI mentions the algorithm, section VII shows simulations and results, and section VIII provides conclusion and future directions in this area.

Standard notation is used where the Euclidean spaces have arbitrary dimensions unless mentioned otherwise; simplification will be done whenever no confusion would arise. We denote the set of real numbers, the *n*-dimensional real vector space, and the set of real *n* × *m* matrices by R, $R^{n}$, and $R^{n\times m}$, respectively. The semi positive definite real space is denoted by $R_{+}$. A comprehensive list of nomenclature is attached at the end of document.

## II Problem formulation and preliminaries

**Fig 1:**
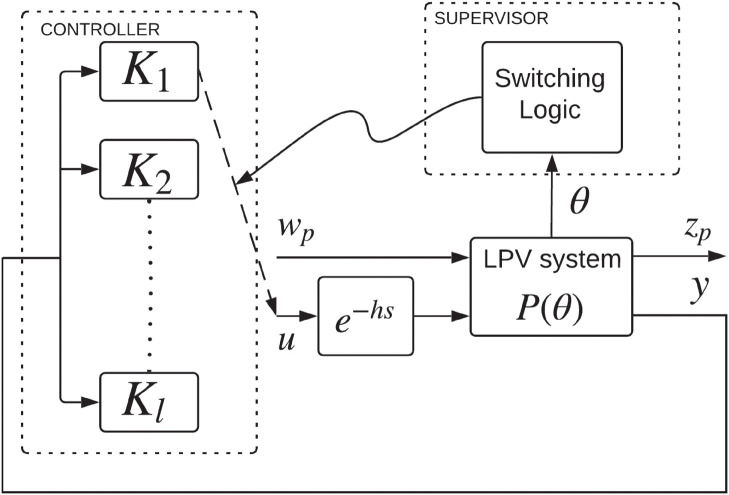
Switching control scheme.

The switching control scheme proposed here is shown in Fig 1. The exogenous input is represented by $w_{p}\in R^{d_{w}}$, $u\in R^{d_{u}}$ shows the control input, the regulated output is shown by $z_{p}\in R^{d_{z}}$, and the measured output is depicted by $y\in R^{d_{y}}$. The constant *h* > 0 represents a delay. The LPV system is dependent on the parameter θ(t)∈R which is presumed to be continuously differentiable. Here, *θ* ∈ Θ, with Θ being a compact set. We build a family of LTI robust controllers which are formulated at specified operating points *θ* = *θ*_*i*_, *i* = 1, 2, …, *l*. We then carry out hysteresis switching between the controllers in compliance to the switching law which is established on the scheduling parameter *θ*(*t*); thus allowing for a larger range of operation for the LPV time delayed system. Our prospect controllers belong to a set $K\overset{▵}{=}\{K_{i}\left(s\right):i=1,2,\ldots ,l\}$; here *K*_*i*_(*s*) is an LTI robust controller constructed for *θ* = *θ*_*i*_.

**Fig 2:**
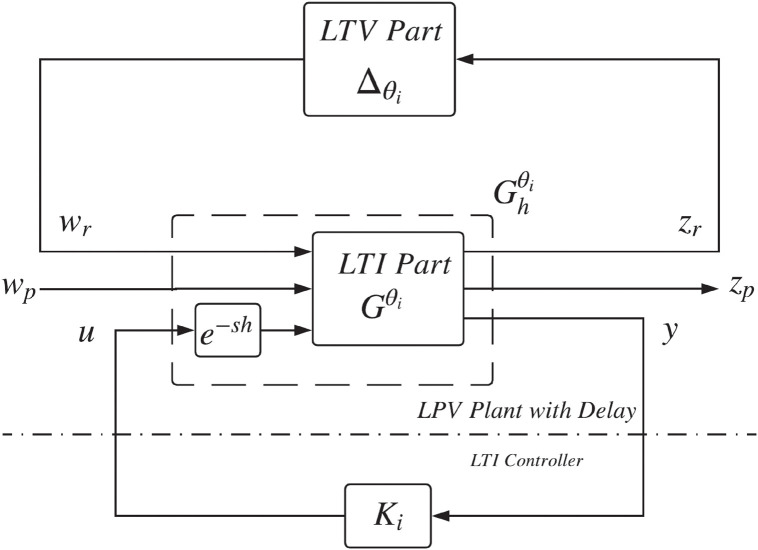
LPV plant with delay and LTI controller.

By considering an operating range Θ_*i*_, *θ*_*i*_ ∈ Θ_*i*_, the LPV time delay system shown in Fig 1 can be represented as $F_{u}\left(G^{\theta_{i}},{△}_{\theta_{i}}\right)$, where $F_{u}$ is the upper linear fractional transformation (LFT), ${△}_{\theta_{i}}$ denotes the time varying part, and $G^{\theta_{i}}$ is the LTI part whose nominal value is *θ*_*i*_. The upper LFT characterization of the closed-loop system of Fig 1 is shown in Fig 2 where the nominal transfer function $G^{\theta_{i}}$ at a specific *θ*_*i*_ is given by

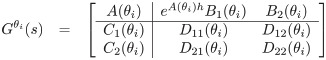
(1)

$$\begin{array}{ccc} & ≜ & \left[\begin{array}{cc} G_{11}^{\theta_{i}}\left(s\right) & G_{12}^{\theta_{i}}\left(s\right)\\ G_{21}^{\theta_{i}}\left(s\right) & G_{22}^{\theta_{i}}\left(s\right) \end{array}\right] \end{array}$$ (2)

where $G_{jk}^{\theta_{i}}\left(s\right)=C_{j}\left(\theta_{i}\right){\left[sI-A\left(\theta_{i}\right)\right]}^{-1}B_{k}\left(\theta_{i}\right)+D_{jk}\left(\theta_{i}\right)$ for *j*, *k* ∈ {1, 2}.

**Assumption 1**. $G_{22}^{\theta_{i}}\left(s\right)$*is stable andD*_22_(*θ*_*i*_) = 0.

Under Assumption 1, the controller *K*_*i*_ in Fig 2 can be chosen based on a Smith predictor [31]. The Smith predictor-based controller consists of a stabilizing compensator $C_{i}$ and a classical Smith predictor Π_*i*_ given by

$$\begin{array}{c} \Pi_{i}\left(s\right)=G_{22}^{\theta_{i}}\left(s\right)-G_{22}^{\theta_{i}}\left(s\right)e^{-sh}\; \end{array}$$ (3)

Remark II.1. If $G_{22}^{\theta_{i}}\left(s\right)$ *is unstable* ($G_{22}^{\theta_{i}}\left(s\right)$ *has both stable and unstable modes) then the controller* *K*_*i*_ *in* Fig 2 *can be chosen as a modified controller based on Smith predictor (unified Smith predictor-based controller)*, [36]. *For the sake of simplicity, we do not propose these extensions*. *D*_22_(*θ*_*i*_) = 0 *implies that the plant* $G^{\theta_{i}}\left(s\right)$ *is strictly proper*.

**Fig 3:**
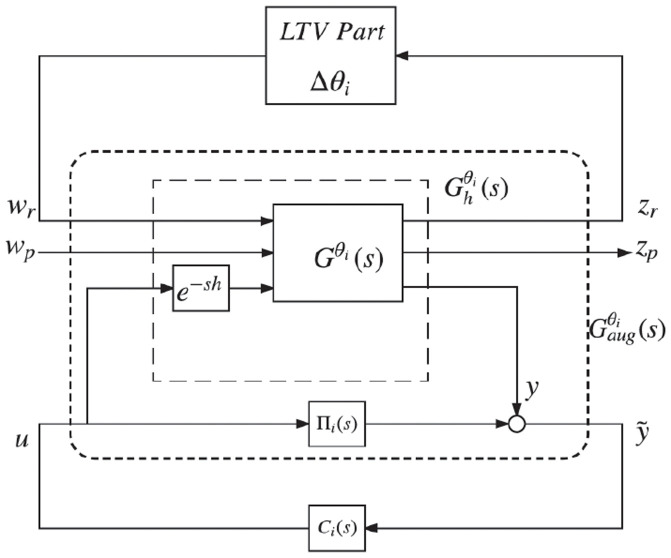
New representation of the system in Fig 2 added with Smith predictor-based controller.

Now we connect the Smith predictor Π_*i*_ in parallel, with the input component *u* to the output *y* of $G_{h}^{\theta_{i}}\left(s\right)$. Hence, we obtain a new representation of the system in Fig 2, now shown in Fig 3, where $\tilde{y}$ is the new output measurement and $G_{aug}^{\theta_{i}}$ is the generalized augmented plant given by

$$\begin{array}{c} G_{aug}^{\theta_{i}}\left(s\right)=[\begin{array}{cc} G_{11}^{\theta_{i}}\left(s\right) & G_{12}^{\theta_{i}}\left(s\right)e^{-sh}\\ G_{21}^{\theta_{i}}\left(s\right) & G_{22}^{\theta_{i}}\left(s\right) \end{array}]\; \end{array}$$

Remark II.2. $C_{i}$ *is a stabilizing controller for* $G_{aug}^{\theta_{i}}$ *iff* $K_{i}=C_{i}{\left(I-\Pi_{i}C_{i}\right)}^{-1}$ *is a stabilizing controller for* $G_{h}^{\theta_{i}}$, [36].

**Fig 4:**
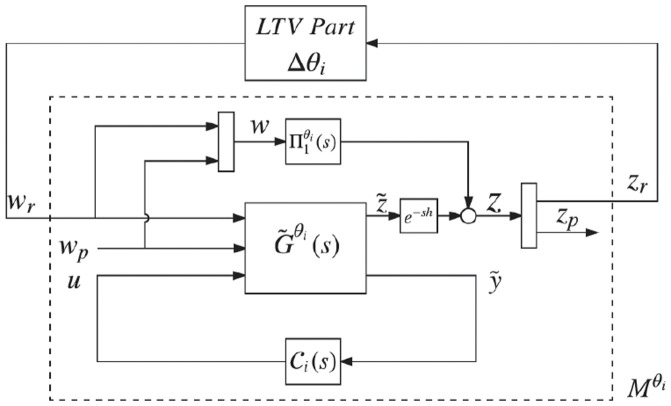
Equivalent representation of the system in Fig 3 by decomposing $G_{aug}^{\theta_{i}}\left(s\right)$.

We obtain an analogous depiction of the system in Fig 3 by decomposing $G_{aug}^{\theta_{i}}\left(s\right)$, [36, Proposition 1], which is shown in Fig 4 where

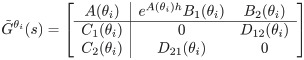
(4)
and

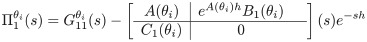


The configuration in Fig 4 will be helpful in solving the standard robust control problem in the next section.

## III Design of robust controllers

Consider the system in Fig 4, where we define an $L_{2}$ optimization problem to find $C_{i}\left(s\right)$ for the LTI system represented by ${\tilde{G}}^{\theta_{i}}\left(s\right)$ in such a way that (i) we ensure the asymptotic stability of the closed-loop system for *θ* ∈ Θ_*i*_, and (ii) inf {sup_*ω*≠0_(‖*z*‖_2_/‖*ω*‖_2_): where $C_{i}\left(s\right)$ is satisfying (i)} ≤ *γ* for the smallest possible value of *γ*, where $z={\left[z_{r}^{T},\;z_{p}^{T}\right]}^{T}$ and $\omega ={\left[\omega_{r}^{T},\;\omega_{p}^{T}\right]}^{T}$. Let ‖.‖_*i*,2_ be the $L_{2}$-induced norm, and define $M^{\theta_{i}}$ in Fig 4 as the transfer function from *ω*_*r*_ to *z*_*r*_. Here, a sufficient condition for robust stability that satisfies (i) is:

$$\begin{array}{c} \|M^{\theta_{i}}{\|}_{\infty }\leq \frac{1}{\|\Delta^{\theta_{i}}{\|}_{i,2}} \end{array}$$ (5)

where

$$\begin{array}{c} M^{\theta_{i}}\left(s\right)=F_{l}\left({\tilde{G}}^{\theta_{i}}\left(s\right),C_{i}\left(s\right)\right)e^{-sh}+\Pi_{1}^{\theta_{i}}\left(s\right) \end{array}$$ (6)

and

$$\begin{array}{c} \begin{array}{c} F_{l}\left({\tilde{G}}^{\theta_{i}}\left(s\right),C_{i}\left(s\right)\right)=\\ {\tilde{G}}_{11}^{\theta_{i}}\left(s\right)+{\tilde{G}}_{12}^{\theta_{i}}\left(s\right)C_{i}\left(s\right){\left[I-{\tilde{G}}_{22}^{\theta_{i}}\left(s\right)C_{i}\left(s\right)\right]}^{-1}{\tilde{G}}_{21}^{\theta_{i}}\left(s\right)\; \end{array} \end{array}$$ (7)

Remark III.1. *The robust stability condition in* (5) *is obtained using small gain theorem reported in* [37].

We deduce that by using (6), the condition (5) is equivalent to

$$\begin{array}{c} \|F_{l}\left({\tilde{G}}^{\theta_{i}}\left(s\right),C_{i}\left(s\right)\right)e^{-sh}+\Pi_{1}^{\theta_{i}}{\left(s\right)\|}_{\infty }\leq \frac{1}{\|\Delta^{\theta_{i}}{\|}_{i,2}}\; \end{array}$$ (8)

Remark III.2. *Note that* $\|\Delta^{\theta_{i}}{\|}_{i,2}<1$ *in* (8) *can be ensured by suitable selection of* $\theta_{i}^{-},\theta_{i}^{+}$ *and* *β*_*i*_ > 0 *such that*,

$$\theta \in \Theta_{i}≔\left[\theta_{i}^{-},\theta_{i}^{+}\right],\;\;\left|\dot{\theta }\left(t\right)\right|<\beta_{i}\;$$

The above treatment leads to robust controller design and the controllers $C_{i}\left(s\right)$ can be synthesized for ${\tilde{G}}^{\theta_{i}}\left(s\right)$ by any standard robust control sysnthesis technique such as mixed sensitivity synthesis method [38] or loop-shaping procedure [39].

Each candidate controller can be described in state space representation as

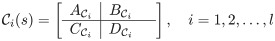
(9)

To cover a larger operating range Θ, a stable switching scheme needs to be developed over $C\overset{▵}{=}\{C_{i}\left(s\right):i=1,2,\ldots ,l\}$. A necessary condition for stable switching is

$$\begin{array}{c} \Theta \subseteq \overset{l}{\underset{i=1}{⋃}}\Theta_{i}\; \end{array}$$ (10)

### III.I Algorithm for design of robust switching controllers

**Fig 5:**
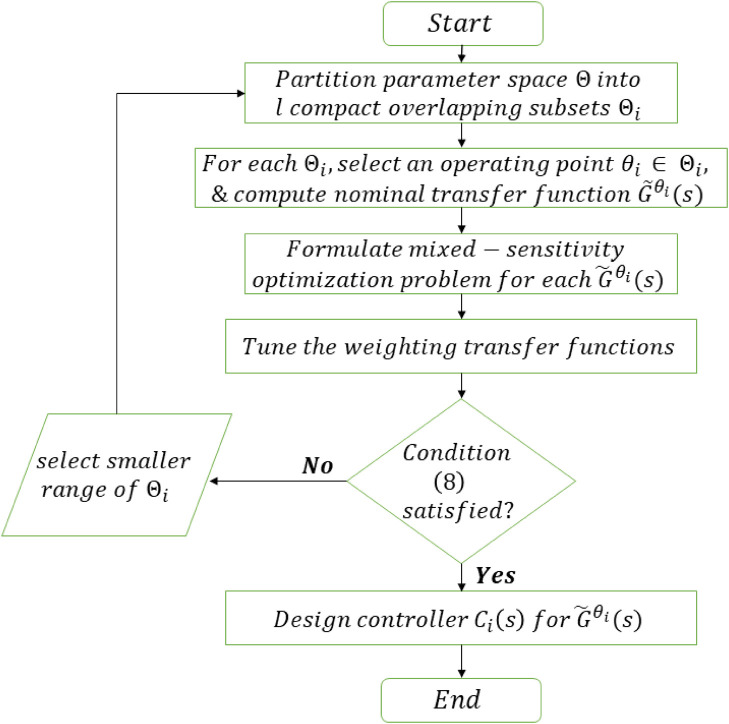
Flowchart of algorithm for design of robust switching controllers.

Here is a brief summary of the design procedure. A summary of the algorithm is shown in Fig 5

**Fig 6:**
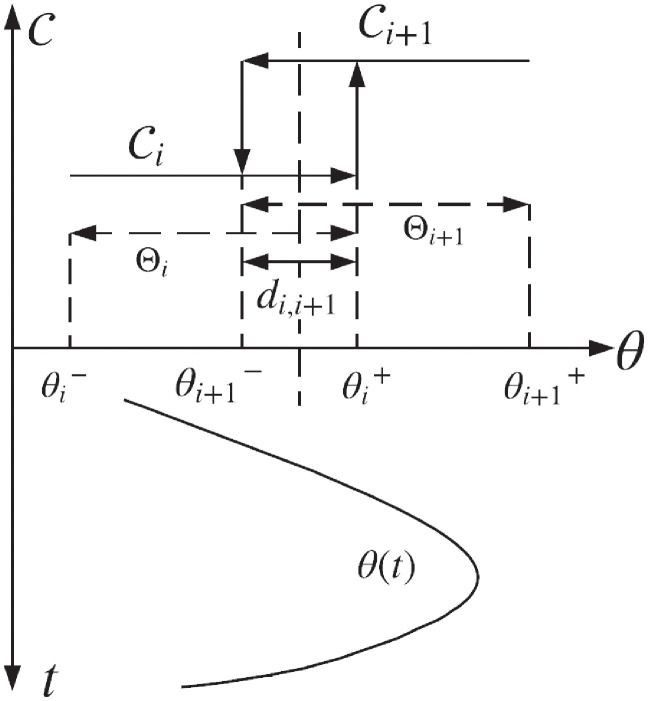
Hysteresis switching.

- Step 1: Partition the parameter space Θ into *l* compact overlapping subsets Θ_*i*_, *i* = 1, 2, …, *l* such that (10) holds true.
- Step 2: For each Θ_*i*_, select an operating point *θ*_*i*_ ∈ Θ_*i*_ and compute the nominal transfer function ${\tilde{G}}^{\theta_{i}}\left(s\right)$ given in (4).
- Step 3: Formulate the mixed-sensitivity optimization problem for each ${\tilde{G}}^{\theta_{i}}\left(s\right)$.
- Step 4: Tune the weighting transfer functions according to the performance specifications and the uncertainty.
- Step 5: Design the controller $C_{i}\left(s\right)$ for ${\tilde{G}}^{\theta_{i}}\left(s\right)$ such that the robust stability criteria (8) is obeyed, and the performance objective from *w*_*p*_ to *z*_*p*_ is achieved. Apply switching according to the hysteresis rule of Fig 6.
- Step 6: If the robust stability condition (8) is not satisfied, then repeat Step 1 to Step 5 with smaller range of Θ_*i*_ and re-tune the weighting transfer functions.

## IV Stability of the closed-loop system

This section provides a sufficient condition for the stability of the closed-loop switched system in terms of dwell time.

Using (4) and (9), the closed-loop matrix A, is given by *A*_*cl*_ ∈ {*A*_*i*_(*θ*), *i* = 1, 2, 3, …, *l*}; here

$$\begin{array}{c} A_{i}\left(\theta \right)=[\begin{array}{cc} A\left(\theta \right)+B_{2}\left(\theta \right)D_{C_{i}}C_{2}\left(\theta \right) & B_{2}\left(\theta \right)C_{C_{i}}\\ B_{C_{i}}C_{2}\left(\theta \right) & A_{C_{i}} \end{array}]\; \end{array}$$ (11)

Consider the switching LPV system here:

$$\begin{array}{c} \dot{\xi }\left(t\right)=A_{q}\left[\theta \left(t\right)\right]\xi \left(t\right),\;t\geq 0 \end{array}$$ (12)

here *q* is a constant signal defined piecewise, whose values belong to set F≔{1,2,…,l}, which essentially means that q(t)=i,i∈F, for ∀*t* ∈ [*t*_*j*_, *t*_*j*+1_). Here $t_{j},j\in Z^{+}\cup \left\{0\right\}$ corresponds to the *j*th switching time point, and $A_{i}\in A≔\left\{A_{i}\left(\theta \left(t\right)\right):i\in F,\theta \left(t\right)\in \Theta \right\}$ represents a family of parameter varying matrices.

**Assumption 2**. *It is assumed that there exists a*

(i) λ_*i*_ > 0; *so that for any* *θ* ∈ Θ, *the eigenvalues of* *A*_*i*_(*θ*) *have real parts that are not greater than* $-2\lambda_{i},\;\forall i\in F$;
(ii) $L_{A}^{i}>0$, *such that*$\|A_{i}\left(\theta \right)\|\leq L_{A}^{i}$, ∀i∈F;
(iii) *there exists a*$L_{D}^{i}>0$*such that*$\|\frac{\partial A_{i}\left(\theta \right)}{\partial \theta }\|\leq L_{D}^{i}$, ∀i∈F;

*here the Euclidean norm of the time-varying vector(which is pointwise in time), and the corresponding induced norm on the matrices are denoted by* ‖.‖.

Consider a family of Lyapunov functions

$$\begin{array}{c} V≔\{V_{i}:V_{i}\left(t,\xi \left(t\right)\right)≔\xi^{T}\left(t\right)Q_{i}\left(t\right)\xi \left(t\right),i\in F\} \end{array}$$ (13)

where *Q*_*i*_(*t*) is a well defined, continuously differentiable, and unique positive-definite solution of

$$\begin{array}{c} {A_{i}}^{T}\left(\theta \left(t\right)\right)Q_{i}\left(t\right)+Q_{i}\left(t\right)A_{i}\left(\theta \left(t\right)\right)=-2\lambda_{i}Q_{i}\left(t\right)-I,\forall i\in F\; \end{array}$$ (14)

There exist positive constants *M*_*i*_ ≥ *μ*_*i*_ > 0; i∈F depends only on λ_*i*_ and $L_{A}^{i}$ such that

$$\begin{array}{c} \mu_{i}{\left\|\xi \left(t\right)\right\|}^{2}\leq V_{i}\left(t,\xi \left(t\right)\right)\leq M_{i}{\left\|\xi \left(t\right)\right\|}^{2},\;t\geq 0\; \end{array}$$ (15)

The reader is refered to [40] for more details. Using Assumption 2 and [40, Lemma 3], we have

$$\begin{array}{c} \|{\dot{Q}}_{i}\left(t\right)\|\leq L_{Q}^{i}\left|\dot{\theta }\left(t\right)\right| \end{array}$$ (16)

where $L_{Q}^{i}>0$ is a constant depending only on λ_*i*_, $L_{A}^{i}$, and $L_{D}^{i}$.

We are now ready to state a sufficient condition in terms of dwell time for stability of the switching LPV system given in (12).

Proposition IV.1. *To obtain hysteresis switching for the set of controllers* C *over the operating range* Θ_*i*_ *satisfying* (10), *a sufficient condition for Lyapunov stability of the switching LPV system* (12) *is*

$$\begin{array}{c} |\dot{\theta }\left(t\right)|<\min\{\min_{i\in F}\{\frac{|d_{i,i+1}|}{h_{D}},\beta_{i}\},\;\beta_{\max}\} \end{array}$$ (17)

where

$$\begin{array}{c} \beta_{i}=\frac{1+2\lambda_{i}\mu_{i}}{L_{Q}^{i}}\;, \end{array}$$ (18)

$$\begin{array}{c} \beta_{\max}=\min_{i\in F}\beta_{i}\;, \end{array}$$ (19)

$$\begin{array}{c} h_{D}=\max_{i\in F}\{\frac{2M_{i}\ln\sqrt{M_{i}/\mu_{i}}}{1+2\lambda_{i}\mu_{i}-L_{Q}^{i}\beta }\}\;,\;0<\beta <\beta_{\max}\;, \end{array}$$ (20)

*and* *d*_*i*,*i*+1_ = Θ_*i*_ ∩ Θ_*i*+1_ *is the ith hysteresis interval as depicted in* Fig 6.

*Proof*. Consider any two neighboring controllers $C_{i}\left(s\right)$ and $C_{i+1}\left(s\right)$ in the time interval [*t*_*j*_ *t*_*j*+*l*_], $j\in Z^{+}\cup \left\{0\right\}$. From the definition of dwell time, i.e., *t*_*j*+1_ − *t*_*j*_ > *h*_*D*_, it follows that the current controller $C_{i}\left(s\right)$ should be active for at least *h*_*D*_. Note that the condition $|\dot{\theta }|<d_{i,i+1}/h_{D}$ is sufficient for stable switching even in the worst case scenario when *θ*(*t*) oscillates around the center of the interval *d*_*i*,*i*+1_; having amplitude |*d*_*i*,*i*+1_|/2 as shown in Fig 6. Considering all possible controllers, and using Remark 3.2 with $|\dot{\theta }\left(t\right)|<\beta_{\max}$ yields (17). This concludes the proof.

Remark IV.1. *It must be noted that* *θ*(*t*) *is assumed as a scalar function of time t*. *When considering the more general case where* $\theta \left(t\right)\in R^{n}$ *is considered to be a vector, we can obtain identical results using similar arguments*. *For the sake of simplicity, we have omitted these results*.

## V Application to blood pressure regulation

In order to demonstrate the efficacy of our results, we consider a practical problem of blood pressure regulation for post-surgical hypertension patients using infusion of vasoactive drug. This problem concerns with the people with impaired built-in autonomic regulation. The control problem deals with regulating mean arterial pressure (MAP) around standard operating point using infusion of vasoactive drug subject to external stimuli in blood pressure. The dynamics of system are parameter-varying due to changes in sensitivity of patients to these vasoactive drugs. The system has an uncertain transport delay, which makes it a reasonable choice for evaluating the performance of our proposed control technique. The importance of this application is highlighted in [41–43]. Note that [29] reports a method to synthesize robust controllers specifically for LPV time delay system that models change in MAP subject to vasoactive drug infusion but it omits stability analysis of switching LPV time delay system.

### V.I Plant description

The plant considered in this paper is an experimentally verified first order time-delayed model, [41, 44–46]. This model describes the change in the MAP subject to injection of the vasoactive drug. The infinite-dimensional transfer function of the setup is given by

$$\begin{array}{c} G\left(s\right)=\frac{\Delta M(s)}{I(s)}=\frac{k}{\tau s+1}e^{-hs} \end{array}$$ (21)

where *k* denotes the sensitivity of the patient to the injected drug given in *mmHg*(*m* *hr*^−1^)^−1^, *τ* is the time for drug distribution, *h* is the transport delay, *I*(*s*) denotes the Laplace transform of the infusion rate of drug in *ml*/*hr*, and Δ*M*(*s*) is the Laplace transform of the relative change in blood pressure (in *mmHg*) from the baseline value of *M*_0_(≃100 *mmHg*), i.e.

$$\begin{array}{c} \Delta M\left(t\right)=M\left(t\right)-M_{0}\; \end{array}$$ (22)

Remark V.1. *We have neglected the recirculation term* *α* *i.e*. *α* ≃ 0 (*in the model of* [44]) *because experimental studies indicate that in most of the cases this recirculation term is not evident*, [41, 45].

Taking the inter-patient and intra-patient variability of response to rate of drug infusion into account, we treat the sensitivity of the patient to the infusion rate as the parameter that varies with time, [41]. Taking state *x*(*t*) = Δ*M*(*t*), input *u*(*t*) = *I*(*t*), time varying parameter *θ*(*t*) = *k*(*t*) and output *y*(*t*) = *M*(*t*), the equivalent LPV state space representation of the system in (21) can be formulated as

$$\begin{array}{c} \begin{array}{ccc} \dot{x}\left(t\right) & = & -\frac{1}{\tau }x\left(t\right)+\frac{\theta (t)}{\tau }u\left(t-h\right)\\ y(t) & = & x\left(t\right)+M_{0}\; \end{array} \end{array}$$ (23)

In the above state space model, we consider the parameter variation *θ*(*t*) ∈ [−9.5, −0.25] *mmHg*(*m* *hr*^−1^)^−1^, [46].

Remark V.2. *We consider* *θ*(*t*) *to be an online measurable parameter and it can be measured online by deploying an extended Kalman filter (EKF) algorithm* [47].

For controller synthesis, we consider the drug distribution time *τ* and the transport delay *h* in (23) to be uncertain with known ranges. The nominal value of *τ* is *τ*_0_ = 35 *sec* with an uncertainty range of [10, 60], and the nominal value of *h* is *h*_0_ = 40 *sec* with an uncertainty range of [20, 60] as mentioned in [46].

### V.II Performance specifications

The aim of the controller is to reduce blood pressure of the patient from a starting value of the 150 *mmHg* to 100 *mmHg* in the presence of time varying parameter *θ*(*t*), uncertainties in *h* and *τ*. The closed-loop control of MAP should fulfill the following performance specifications, [44, 46].

1. The settling time needs to be less than 10 minutes.
2. The pressure undershoot; in other words the peak hop below the desired level, must be less than 10 mmHg.
3. The MAP should be inside ±5 *mmHg* of the desired set-point during the steady state.
4. Unstable modes and oscillatory response are unacceptable at any time.
5. Vasoactive drug infusion rate should be bounded as 0 < *I*(*t*) < 180 *ml* *hr*^−1^ (for Sodium Nitroprusside) to avoid the side effects.

### V.III Controller synthesis

For the synthesis of *ith* controller, we consider the following uncertain plant

$$\begin{array}{c} \begin{array}{c} G_{i}\left(s\right)=\frac{\left(\theta_{i}+\Delta_{i}\right)e^{(-h_{0}+\Delta h)s}}{(\tau_{0}+\Delta \tau )s+1},\;\text{for}\;i=1,2,\ldots ,l \end{array} \end{array}$$ (24)

where $\Delta_{i}=\left|\theta_{i}^{+}-\theta_{i}^{-}\right|$, Δ*h* = 20, and Δ*τ* = 25. The uncertain plant can be modeled as $G_{i}\left(s\right)=G_{0_{i}}\left(s\right)+G_{\Delta_{i}}\left(s\right)$, for i=1,2,…,l where $G_{0_{i}}\left(s\right)$ is a nominal version of the plant in (23) evaluated at *θ* = *θ*_*i*_ and a bound on $G_{\Delta_{i}}\left(s\right)$ can be chosen as

$$\begin{array}{c} \begin{array}{c} \left|G_{\Delta_{i}}\left(s\right)\right|\leq \left|\Delta_{i}\right|+|\theta_{i}|\left(\right|e^{-\Delta hs}-1|+\\ |\Delta \tau \frac{s}{\tau_{0}s+1}\left|)\leq \right|\Delta_{i}|+2.715\times \left|\theta_{i}\right| \end{array} \end{array}$$ (25)

for *i* = 1, 2, …, *l*.

To satisfy the robust stability condition (8) and to fulfill performance specifications listed in Section V.II, we partition the operating region of the plant in (23) into five smaller operating regions (*l* = 5) given below.

$$\begin{array}{c} \begin{array}{c} \Theta_{1}=\left[\theta_{1}^{-},\;\theta_{1}^{+}\right]=\left[-9.50,\;-5.80\right]\;\text{for}\;\text{controller}\;C_{1};\\ \Theta_{2}=\left[\theta_{2}^{-},\;\theta_{2}^{+}\right]=\left[-5.80,\;-3.03\right]\;\text{for}\;\text{controller}\;C_{2};\\ \Theta_{3}=\left[\theta_{3}^{-},\;\theta_{3}^{+}\right]=\left[-3.03,\;-1.42\right]\;\text{for}\;\text{controller}\;C_{3};\\ \Theta_{4}=\left[\theta_{4}^{-},\;\theta_{4}^{+}\right]=\left[-1.42,\;-0.60\right]\;\text{for}\;\text{controller}\;C_{4};\\ \Theta_{5}=\left[\theta_{5}^{-},\;\theta_{5}^{+}\right]=\left[-0.60,\;-0.25\right]\;\text{for}\;\text{controller}\;C_{5}. \end{array} \end{array}$$

Remark V.3. *A single controller cannot satisfy the robust stability condition* (8) *and the performance specifications listed in Section V.II due to large range of uncertainty in the system* 23.

A family of five robust controllers is constructed with each controller corresponding to a single operating range mentioned above. The LTI controllers $\left\{C_{i}\left(s\right):i=1,2,\ldots ,5\right\}$ are designed for specific operating points, i.e., for corresponding values of *θ* as: *θ* = *θ*_1_ = −7.65, *θ* = *θ*_2_ = −4.415, *θ* = *θ*_3_ = −2.225, *θ* = *θ*_4_ = −1.01, and *θ* = *θ*_5_ = −0.425, respectively. To avoid chattering, hysteresis switching as shown in Fig 6 is employed among the family of five controllers. The hysteresis based supervisory controller switching logic is given in Table 1. The table provides the thresholds on the values of parameter on which the controllers will be switching.

**Table 1:** Hysteresis based supervisory controller logic.

Switching Logic	@ Value of *θ*(*t*)
Switch: $C_{1}\to C_{2}$	@ *θ* = −5.60
Switch: $C_{1}\leftarrow C_{2}$	@ *θ* = −6.00
Switch: $C_{2}\to C_{3}$	@ *θ* = −2.83
Switch: $C_{2}\leftarrow C_{3}$	@ *θ* = −3.23
Switch: $C_{3}\to C_{4}$	@ *θ* = −1.22
Switch: $C_{3}\leftarrow C_{4}$	@ *θ* = −1.62
Switch: $C_{4}\to C_{5}$	@ *θ* = −0.40
Switch: $C_{4}\leftarrow C_{5}$	@ *θ* = −0.80

We use the standard two-block mixed sensitivity synthesis method to design the LTI robust controller $C_{i}$ following the treatment developed in previous sections. Sensitivity and control sensitivity weighing functions have been incorporated for the controller design. For reference tracking, an additional constraint on $C_{i}$ i.e., $\lim_{s\to 0}C_{i}{\left(I+\Pi_{i}C_{i}\right)}^{-1}=\infty$, is considered. For instance, the sensitivity weight $W_{s_{1}}$ and the control sensitivity weight $W_{k_{1}}$ corresponding to the operating range $\Theta_{1}=\left[-9.5,\;-5.80\right]$ to fulfill the performance specifications are selected as

$$\begin{array}{c} \begin{array}{ccc} W_{s}\left(s\right) & = & \exp\left(\frac{-h}{\tau_{0}}\right)\times \frac{s+0.055}{10s+5.5\times 10^{-4}}\\ W_{k} & = & \exp\left(\frac{-h}{\tau_{0}}\right)\times \left(\right|\Delta_{1}\left|+2.715\times \right|\theta_{1}\left|\right)=7.2118\; \end{array} \end{array}$$ (26)

Then we solve the standard two block mixed sensitivity synthesis problem using MATLAB for the operating range Θ_1_ = [-9.5, -5.80] which satisfies the robust stability condition (8) with *γ* = 0.9443. A similar procedure is adopted for the synthesis of the controllers $C_{2}$ through $C_{5}$. We have separately simulated the response for $C_{3}$ (referred as single controller) for performance comparison of switching and non-switching control.

## VI Results and discussions

**Fig 7:**
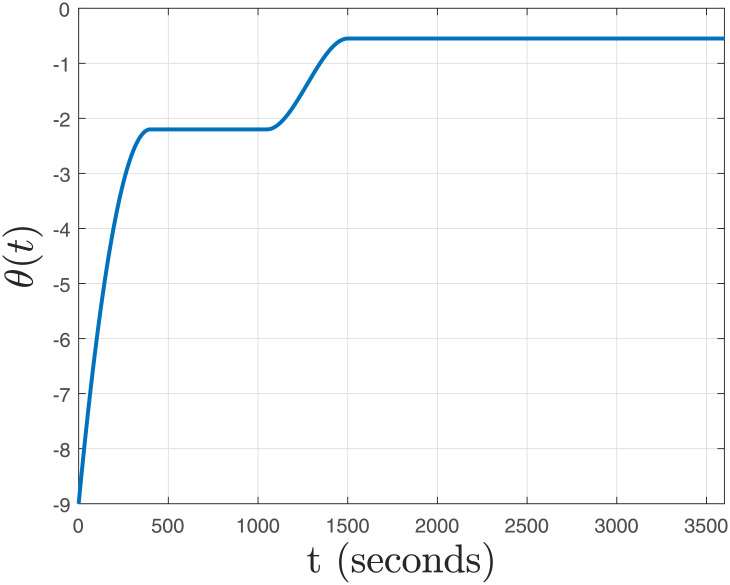
Time varying parameter, *θ*(*t*).

**Fig 8:**
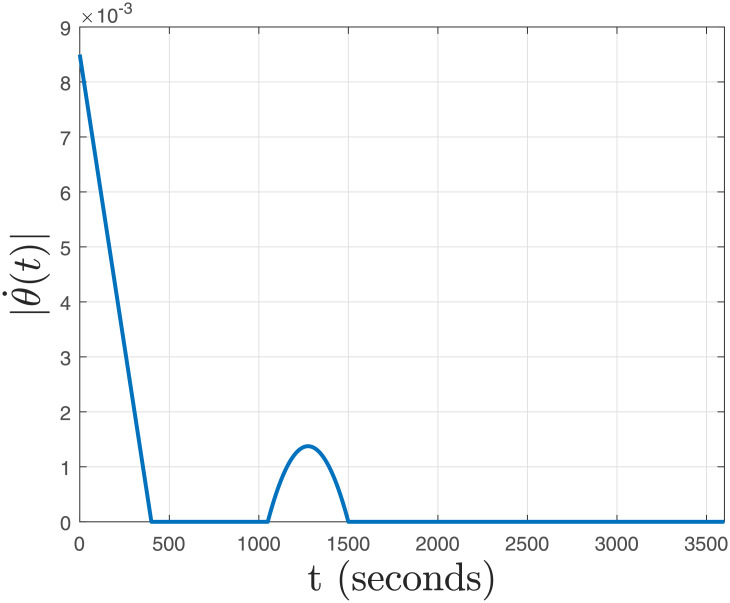
Rate of parameter variation, $|\dot{\theta }\left(t\right)|$.

**Fig 9:**
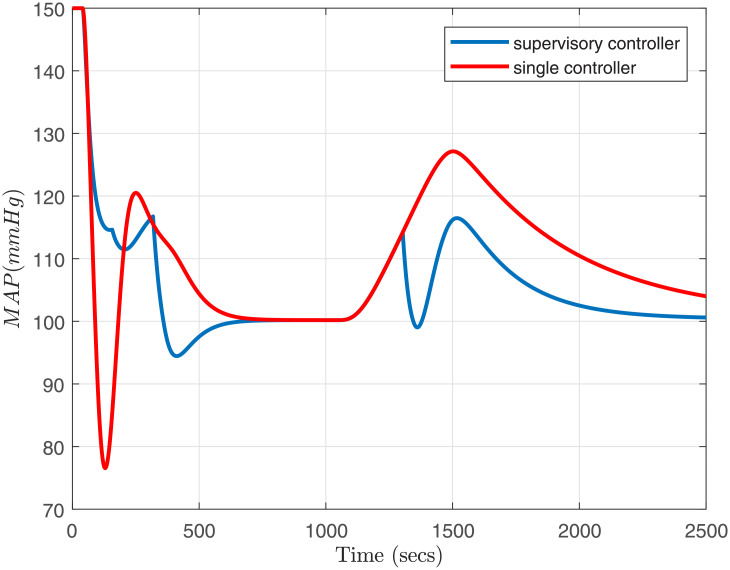
Curve of mean arterial pressure, Δ*M*(*t*).

**Fig 10:**
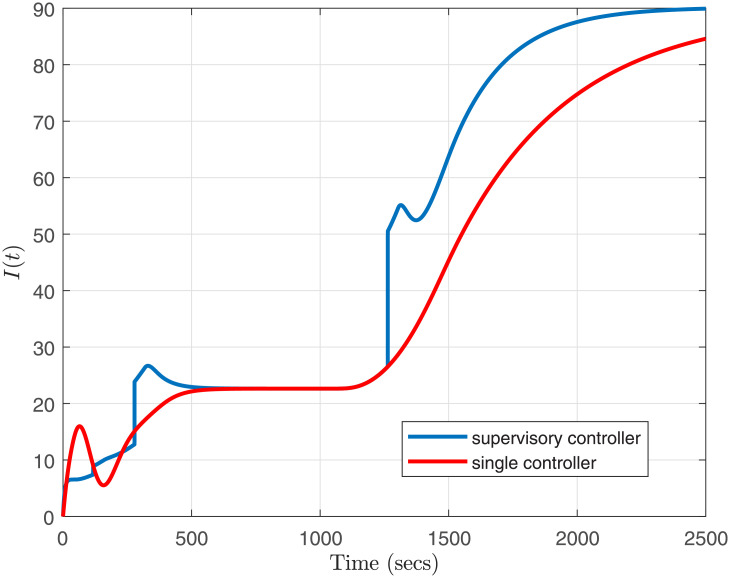
Curve of drug infusion rate, *I*(*t*).

This section includes the simulation results and discussion for the closed-loop switching LPV system formulated previously. The simulations were carried out using MATLAB 2016a on a Lenovo ThinkPad Machine X220i, which has an Intel Core i3 processor with a RAM of 8 GB. The simulation results are shown in Figs 7–10. The parameter trajectory *θ*(*t*) is shown in Fig 7 whose range was descibed in [46]. As seen in the plot, *θ*(*t*) rises during time intervals [0, 400] and [1100, 1400]; while it remains constant during the interval [400, 1100] and for time ≥ 1400 seconds. *θ*(*t*) is the signal used by the supervisory controller for switching in between the controllers. The parameter trajectory includes the entire operating range of LPV system, and this choice is made solely to validate the performance of our proposed method in the worst-case scenarios.

The absolute rate of parameter variation $|\dot{\theta }\left(t\right)|$ is shown in Fig 8. Using Proposition IV.I, we have *β*_max_ = 0.0279 and *h*_*D*_ = 472.71. From Fig 8, we have $\sup_{\forall t}\left|\dot{\theta }\left(t\right)\right|=0.0085<\beta_{\max}=0.0279$; which ensures Lyapunov stability in the presence of both uncertainity, and delay under the parameter trajectory given in Fig 7.

We have used constant Lyapunov matrix and solved the LMIs from Eq (14) on the vertices of the polytope for each operating region. The closed-loop system is simulated using the family of five switching robust controllers, $\left\{C_{i}\left(s\right):i=1,2,\ldots ,5\right\}$. Bump-less transfer is pre-conditioning ensured for all the controllers before the simulation. For implementation of the Smith-predictor structure, an FIR filter has been used as in [29]. Fig 9 compares the response of *MAP* under parametric variations and admissible switching scheme with a single conroller. As seen in 9, the supervisory approach satisfies all the required performance specifications simultaneously, i.e., (*i*) settling time is less than 10 minutes, (*ii*) pressure undershoot was maintained less than 10 mmHg, and (*iii*) Mean Arterial Pressure (MAP) is within ±5 *mmHg* of the desired set-point. In comparison, a single controller fails to secure these specifications simultaneously. However, the infusion rate *I*(*t*) is within the desired limits of 0 < *I*(*t*) < 180 *ml* *hr*^−1^ for both controller structures as evident from Fig 10.

Our technique circumvents Padé approximation and as shown in the simulations above, it yields satisfactory performance for larger range of parameter variation and uncertainty as compared to [41]. Hence, the simulation results show that the control structure designed here conforms with our derivations from the previous sections.

## VII Conclusion

A deterministic approach is proposed in this paper for supervisory control of LPV time delay systems. A family of Smith predictor based robust controllers is synthesized where each controller ensures robust stability in the neighborhood of pre-selected operating points in the presence of delay. Under hysteresis switching, we provided a sufficient condition for Lyapunov stability of the closed-loop LPV system in terms of bound on the rate of parametric variations. The approach is quite effective for LPV time delay systems with slow variations in parameters. Moreover, the operating range for the system is stretched by utilizing this approach. The approach doesn’t consider Lyapunov-Krasovskii functionals which makes it more tractable, less conservative and computationally cheap. The resulting controller are easy to implement in practical applications. The efficacy of the switching robust control scheme is illustrated by applying it to blood pressure regulation of postsurgical hypertension patients.

Some future extensions include finding stability conditions for LPV systems with time-varying delays and derivation for less conservative bound on parameter variation to allow faster switching.

## Supporting information

S1 Nomenclature (PDF)
