# Supplementary material for: A deterministic approach for design of supervisory control of LPV systems with delay
Source: PLoS One. 2021 Aug 20;16(8):e0256408. doi: 10.1371/journal.pone.0256408 (PMC8378726; doi:10.1371/journal.pone.0256408)
Supplement: S1 Nomenclature — (PDF) [file pone.0256408.s001.pdf]

Nomenclature

$\mathbb{R}$	Set of real numbers
$\mathbb{R}_+$	Semi-positive definite real space
w_p	Exogenous input
u	Control input
z_p	Regulated output
y	Measured output
$h(h_0)$	Time-delay(nominal value)
$\theta(t)$	Scheduling parameter
Θ	Compact set
$K_i(s)$	LTI robust controller designed for $\theta = \theta_i$
Θ_i	Operating range
$\mathcal{F}_u$	Upper linear fractional transformation (LFT)
$\triangle_{\theta_i}$	Time-Varying part of the LPV system
G^{θ_i}	LTI part with nominal value θ_i
$\mathcal{C}_i$	Stabilizing compensator(Smith predictor)
Π_i	Classical Smith predictor
$G^{\theta_i}_{aug}$	Generalized augmented plant
A_{cl}	Closed-loop A-matrix
q	Piecewise constant signal
$\mathcal{A}$	Family of parameter-varying matrices
$\|\|.\|\|$	Euclidean norm
$\mathscr{V}$	Family of Lyapunov functions
$\mathfrak{C}$	Controller set
$G(s)$	Infinite-dimensional transfer function
k	Patient's Sensitivity to injected drug$(mmHg(m\ hr^{-1})^{-1})$
$\tau(\tau_0)$	Drug distribution time(nominal value)
$I(s)$	Laplace transform of drug infusion rate(ml/hr)
$\Delta M(s)$	Laplace transform of relative change in blood pressure
M_0	Baseline value
$G_i(s)$	Uncertain plant
$G_{0_i}(s)$	Nominal plant
W_s	Sensitivity weight
W_k	Control sensitivity weight
$\|\dot{\theta}(t)\|$	Absolute rate of parameter variation
